# The Use of Artificial Intelligence in Gastroenterology: A Glimpse Into the Present

**DOI:** 10.14309/ctg.0000000000000653

**Published:** 2023-10-20

**Authors:** Brian C. Jacobson

**Affiliations:** 1 Massachusetts General Hospital, Boston, Massachusetts, USA.

“Doctor doesn’t use his head and education to figure out what’s the matter with you. Machines go over you—measure this, measure that. Then he picks out the right miracle stuff, and the only reason he does is on account of the machines tell him that’s what to do.”

—Kurt Vonnegut, *Player Piano*, 1952

Artificial Intelligence (AI) is not the future, it's the present. More accurately, it's the past, though new breakthroughs have raised it to the forefront of the public's awareness. Machine learning, where computers can mine vast quantities of data seeking predictive patterns, offers the potential for new screening and diagnostic methods. Artificial neural networks, a form of machine learning, seeks to mimic human brain function in the sense that our neurons signal to each other, taking a stimulus and linking it to specific outputs (think about how you know it's a hairbrush when you look at a hairbrush or you remember some childhood memory when you smell a specific odor). Despite neither using nor needing a hairbrush, a computer can be taught (or even self-learn) to recognize one. Now suppose the question is not “is this a hairbrush?” but rather “is this an adenoma?” or “what is this person's risk of harboring esophageal cancer?”

This month's issue of *Clinical and Translational Gastroenterology* is devoted to various ways in which AI is being applied to improve the care of patients with gastrointestinal conditions. When I think about AI, I am struck by its potential to enhance both the care we deliver and the additional time we may save while providing that care. In this special issue, you'll find examples of how AI may be able to improve diagnostic accuracy, whether it's computer-aided diagnosis of *in situ* colon polyps (1), real-time detection of *Helicobacter pylori* infection (2), or determining the invasion depth of esophageal squamous cell carcinoma (3). AI can also improve patient access to GI diagnostics, as some technologies may become more easily utilized by primary care providers or allied health professionals. To that end, we present data on AI's ability to diagnose gastric lesions using only capsule endoscopy (4) and as a tool to render a diagnosis during anorectal manometry (5).

Another exciting application of AI is its ability to improve population health screening. In this month's issue, you'll read about machine learning's utility in searching through electronic health records to predict who may develop incident Barrett's esophagus and esophageal adenocarcinoma (6) and to review cross-sectional imaging to identify patients with cirrhosis (7). In addition, the promise of AI to save us time in our daily activities is also addressed. For example, AI may be useful in reading our patients' pH and impedance studies (8) or providing a diagnosis of subepithelial gastric lesions without having to perform fine needle biopsies (9).

However, as we embrace AI-assisted health care, there is also a need for caution. We must remain extraordinarily vigilant in how AI interprets data, lest we perpetuate many of the biases, inequities and disparities already known to plague our current healthcare system. Much has been written about the problem of “garbage in, garbage out” that can impact AI systems and researchers have appropriately begun paying careful attention to, and safeguarding against, algorithmic discrimination. Some have even called for studying biases in AI-rendered data as a means of better detecting and understanding the population inequities present in healthcare (10).

With all this in mind, I hope you find this month's issue both fascinating and inspiring. We stand on the threshold of a new era in healthcare delivery; one in which computers disrupt our traditional methods of caring for patients. Vonnegut's writing was indeed prophetic. It's up to us to make sure our patients reap the benefits, and not the risks, promised by this tremendous technological advance.

## CONFLICTS OF INTEREST

**Guarantors of the article:** Brian C. Jacobson, MD, MPH, FACG.

**Specific author contributions:** sole author.

**Financial support:** None to report.

**Potential competing interests:** None

## References

[1] El Zoghbi M Shaukat A Hassan C Artificial intelligence-assisted optical diagnosis: A comprehensive review of its role in leave-in-situ and resect-and-discard strategies in colonoscopy. Clin Transl Gastroenterol 2023. doi. 10.14309/ctg.0000000000000640 PMC10584286 37747097

[2] Shen Y Chen A Zhang X Real-time evaluation of Helicobacter pylori infection by convolution neural network during white-light endoscopy: A prospective, multicenter study. Clin Transl Gastroenterol 2023. doi. 10.14309/ctg.0000000000000643 PMC10589579 37800683

[3] Zhang L Luo R Tang D Human-like artificial intelligent system for predicting invasion depth of esophageal squamous cell carcinoma using magnifying narrow-band imaging endoscopy: A retrospective multicenter study. Clin Transl Gastroenterol 2023. doi. 10.14309/ctg.0000000000000606 PMC10589558 37289447

[4] Mascarenhas M Mendes F Ribeiro T Deep learning and minimally invasive endoscopy: Automatic classification of pleomorphic gastric lesions in capsule endoscopy. Clin Transl Gastroenterol 2023. doi. 10.14309/ctg.0000000000000609 PMC10584281 37404050

[5] Saraiva MM Pouca MV Ribeiro T Artificial intelligence and anorectal manometry: Automatic detection and differentiation of anorectal motility patterns - a proof of concept study. Clin Transl Gastroenterol 2022. doi. 10.14309/ctg.0000000000000555 PMC10584284 36520781

[6] Iyer PG Sachdeva K Leggett CL Development of electronic health record based machine learning models to predict barrett's esophagus and esophageal adenocarcinoma risk. Clin Transl Gastroenterol 2023. doi. 10.14309/ctg.0000000000000637 PMC10584285 37698203

[7] Mazumder NR Enchakalody B Zhang P . Using artificial intelligence to predict cirrhosis from CT scans. Clin Transl Gastroenterol 2023. doi. 10.14309/ctg.0000000000000616 PMC10584300 37436183

[8] Zhou MJ Zikos T Goel K Development and validation of a machine learning system to identify reflux events in esophageal 24-hour pH/impedance studies. Clin Transl Gastroenterol 2023. doi. 10.14309/ctg.0000000000000634 PMC10584295 37578060

[9] Zhu C Hua Y Zhang M . A multimodal multipath artificial intelligence system for diagnosing gastric protruded lesions on endoscopy and endoscopic ultrasonography images. Clin Transl Gastroenterol 2022. doi. 10.14309/ctg.0000000000000551 PMC10584296 36434804

[10] Ferryman K Mackintosh M Ghassemi M. Considering biased data as informative artifacts in AI-assisted health care. N Engl J Med 2023;389:833–8. 37646680 10.1056/NEJMra2214964

